# Face Processing in Early Development: A Systematic Review of Behavioral Studies and Considerations in Times of COVID-19 Pandemic

**DOI:** 10.3389/fpsyg.2022.778247

**Published:** 2022-02-18

**Authors:** Laura Carnevali, Anna Gui, Emily J. H. Jones, Teresa Farroni

**Affiliations:** ^1^Department of Developmental Psychology and Socialization, University of Padua, Padua, Italy; ^2^Centre for Brain and Cognitive Development, Birkbeck, University of London, London, United Kingdom

**Keywords:** face processing, development, infancy, social cognition, mask wearing, COVID-19

## Abstract

Human faces are one of the most prominent stimuli in the visual environment of young infants and convey critical information for the development of social cognition. During the COVID-19 pandemic, mask wearing has become a common practice outside the home environment. With masks covering nose and mouth regions, the facial cues available to the infant are impoverished. The impact of these changes on development is unknown but is critical to debates around mask mandates in early childhood settings. As infants grow, they increasingly interact with a broader range of familiar and unfamiliar people outside the home; in these settings, mask wearing could possibly influence social development. In order to generate hypotheses about the effects of mask wearing on infant social development, in the present work, we systematically review *N* = 129 studies selected based on the most recent PRISMA guidelines providing a state-of-the-art framework of behavioral studies investigating face processing in early infancy. We focused on identifying sensitive periods during which being exposed to specific facial features or to the entire face configuration has been found to be important for the development of perceptive and socio-communicative skills. For perceptive skills, infants gradually learn to analyze the eyes or the gaze direction within the context of the entire face configuration. This contributes to identity recognition as well as emotional expression discrimination. For socio-communicative skills, direct gaze and emotional facial expressions are crucial for attention engagement while eye-gaze cuing is important for joint attention. Moreover, attention to the mouth is particularly relevant for speech learning. We discuss possible implications of the exposure to masked faces for developmental needs and functions. Providing groundwork for further research, we encourage the investigation of the consequences of mask wearing for infants’ perceptive and socio-communicative development, suggesting new directions within the research field.

## Introduction

Faces are our primary source of information about other people. We rely on social cues conveyed by human faces to interpret socio-communicative interactions, using information from the face to decode others’ intentions, emotions, and interests. Since the early stages of the COVID-19 pandemic, the World Health Organization (WHO) recommended wearing face masks in social contexts to limit viral diffusion (WHO, 2020). This brings an important change in the facial information available for encoding, leaving eyes uncovered while masking the mouth. Face coverings remove information about facial configuration and potentially affect social cognition by altering face perception and detection of communicative meanings in social contexts in adults (Carragher and Hancock, 2020; Noyes et al., 2021) and school-aged children (Stajduhar et al., 2021). Considering the effects of face coverings on social cognition is important in evaluating the risk–benefit balance of mask mandates in particular settings.

The roots of social cognition begin at birth and critically rely on processing information from faces. Newborns preferentially orient toward faces (Morton and Johnson, 1991; Gamé et al., 2003; Macchi Cassia et al., 2004), an effect driven by the configural location of the eyes and mouth (Morton and Johnson, 1991; Farroni et al., 2005). The most frequent stimulus infants encounter in their environment is the human face (Fausey et al., 2016). Being exposed to a variety of facial features (eyes, eye gaze, and mouth) and emotional expressions within sensitive periods is crucial for the specialization of social brain networks (Johnson, 2005). Thus, given that masks disrupt visual access to facial features, it is important to consider the possible cascading effects of exposure to masked faces on perceptive and socio-communicative development. Since a large corpus of published literature has examined how early exposure to faces contributes to social brain development, we can leverage existing work to ask which aspects of face processing may be altered by exposure to masked faces and whether this has different implications depending on one’ s developmental stage.

In the present paper, we summarize the wide corpus of studies on the development of face processing to understand possible effects of mask wearing as a function of infants’ developmental needs. To generate hypotheses, we consider the changes in facial cues resulting from mask wearing (mouth covered and eyes uncovered and breaking face configuration) and present a guided systematic review of behavioral studies investigating face processing during the first years of life (0–36 months). Mask wearing is discussed in terms of both altering face perception and hindering social communication by removing information about face configuration. Crucially, the aim of this review is to inform future research exploring the developmental effects of mask wearing, which is a key preventive measure to limit COVID-19 diffusion.

## Materials and Methods

Two literature searches were conducted on Elsevier’s Scopus^®^ (Ballew, 2009) and OVID databases before February 20th, 2021 to select papers on the topic of face processing in infancy. The search string was {[(face and (face processing or eye or eyes or mouth or gaze or emotion or motion or race) and infan*) not (“autism spectrum disorders” or asd or asc or autis* or ndd or “neurodevelopmental disorde*”)].ti,ab,kw.} yielded 8,828 manuscripts in total. Manuscripts were selected from subject areas of Psychology, Neuroscience and Social Sciences as published or in press articles written in English; then, duplicates were removed resulting in 5155 papers to be screened. We focused on behavioral studies with typically developing infants to get a sense of possible observed effects of mask mandates in community contexts for children in preschool age.

An additional automatic filter was applied before manual abstract screening, such that the retrieved manuscripts’ title, abstract, or keywords had (1) to include or (2) not to include words as indicated in Table 1. This strategy was adopted to limit the search to content which was pertinent to our research questions. Two independent researchers (LC and AG) screened the remaining abstracts (*N* = 615) and read all the selected papers (*N* = 110). By reading abstracts, papers were excluded if non-relevant in terms of topic, age, non-behavioral methodology (EEG, NIRS, fMRI, and pupillary reflex), publication type being a review, or meta-analysis, publication date before 2000. Each of the selected papers was assigned to one or more from the following topics: “eyes,” “gaze cueing,” “mouth,” “motion,” “local/global,” “emotion,” “race,” and “face looking.” To limit the focus of this review to the effect of facial features and information that could be altered or hidden by masks, papers focusing on the effect of race on face perception were excluded at this stage.

**Table 1:** Criteria used for manuscript search.

String	Keywords	
	Limited to	Excluded
{[(face and (face processing or eye or eyes or mouth or gaze or emotion or motion or race) and infan*) not (“autism spectrum disorders” or asd or asc or autis* or ndd or “neurodevelopmental disorde*”)].ti,ab,kw.}	Newborn, Infant, Child, Preschool Child, Child, Preschool, Face, Facial Expression, Emotion, Mouth, Attention, Child Development, Emotions, Child Behavior, Infancy, Psychology, Information Processing, Visual Perception, Gaze, Perception, Pattern Recognition, Visual, Eye Movement, Nose, Facial Recognition, Recognition, Social Behavior, Fixation, Ocular, Eye Fixation, Eye-Tracking, Eye, Face Processing, Infant Behavior, Social Interaction	Adolescent, School Child, Middle Aged, Major Clinical Study, Temperament, Pregnancy, Animal, Prematurity, Autistic Disorder Autism Spectrum Disorder, Clinical Feature, Sex Difference, Comparative Study, Animals, Aging, Photostimulation, Neuroimaging, Electroencephalography, Evoked Response, Nuclear Magnetic Resonance Imaging, Pathophysiology, Magnetic Resonance Imaging, Electroencephalogram, Hemispheric Dominance, Evoked Potentials

**Figure 1:**
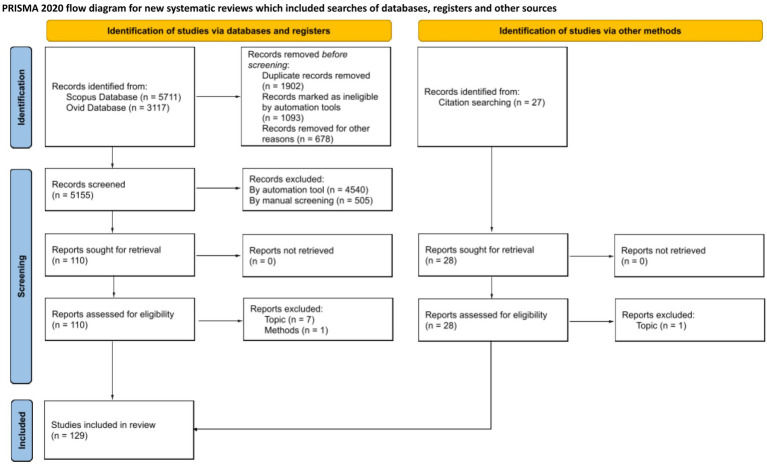
PRISMA flow diagram.

Selection bias could possibly happen based on automation tool selection; however, we attempted to overcome this by carefully selecting relevant references during full-text reading. An additional *N* = 28 papers were manually included at this stage. Nine papers were excluded after full-text reading as considered out of topic. The final sample was *N* = 129 papers. The literature selection process is illustrated in the PRISMA flow diagram (Figure 1).

## Results

Papers included in the present review covered five main topics: face exposure (i), featural and configural face scanning (ii), eye and eye gaze (iii), mouth (iv), and emotion expression (v). These topics were selected to allow us to extrapolate the possible impact of mask wearing linked to: being exposed to a smaller variety of uncovered faces and possibly to familiar faces more often than before the COVID-19 pandemic (i), being exposed to partly covered faces rather than full faces (ii), having the eyes region uncovered and available to extract information (iii), obtaining limited information from the mouth and mouth movements (iv), and having limited experience with simultaneous changes in face features due to emotional expressions (v). Importantly, perceptual and communicative aspects are examined in each paragraph. In Table 2, we provide a summary of the main information for each included study.

**Table 2:** Summary of studies included in the review.

Authors	Topic(s)	Age (months)	Method	Eye tracker	Sample size
Aktar et al., 2018	Emotion	14	Free-viewing task	Yes	*n* = 57 (23F)
Amano et al., 2004	Motion	3–4	Live interaction	No	Exp 1: *n* = 24 (10F), Exp 2: *n* = 22 (13F)
Atagi and Johnson, 2020	Mouth	15–25	Free-viewing task	Yes	*n* = 77 of which *n* = 34 (11F; monolinguals) *n* = 43 (15F; bilinguals)
Bahrick and Newell, 2008	Motion	5.5	Familiarization	No	Exp 1: *n* = 24 (13F), Exp 2: *n* = 24 (10F)
Bahrick et al., 2013	Mouth	2 and 3	Habituation	No	Exp 1: *n* = 48 (15F, 2 months) Exp 2: *n* = 32 (17F, 3 months)
Bhatt et al., 2005	Featural/configural	3, 5	Habituation	No	Exp 1: *n* = 28 3 m (13F) Exp 2: *n* = 32 of which *n* = 16 3 m (8F) *n* = 16 5 m (7F) Exp 3: *n* = 16 3 m (8F) Exp 4: *n* = 32 5 m (18F)
Brenna et al., 2013	Emotion	3	Familiarization	No	*n* = 64 (26F)
Brooks and Meltzoff, 2002	Eyes	12, 14, and 18	Live interaction	No	Exp 1: *n* = 96 Exp 2: *n* = 96
Cashon et al., 2013	Featural/configural, Face exposure	22–25 weeks 27–32 weeks	Habituation	No	*n* = 111 (42F)
Cecchini et al., 2011	Motion, Featural/configural, Face exposure	Newborns	Familiarization	No	*n* = 16
Chen et al., 2020	Gaze cuing	12–37	Free play	Yes	*n* = 21 12–37 m
Chien et al., 2010	Featural/configural	2–4.5	Forced-choice novelty preference	No	*n* = 24
Cohen and Cashon, 2001	Featural/configural	7	Habituation	No	*n* = 32 (16F)
Coulon et al., 2011	Mouth, motion	Birth	Familiarization	No	Exp 1: 16 (7F) Exp 2: 16 (9F)
Courage et al., 2006	Face exposure	3,5–13	Free-viewing task	No	*n* = 100 (50F)
de Haan et al., 2004	Emotion	7	Video coding looking time	No	*n* = 40 (11F)
de Heering et al., 2008	Face exposure	Birth	Habituation	No	*n* = 28
de Heering et al., 2015	Featural/configural	3	Preferential looking	No	*n* = 40 (23F)
Della Longa et al., 2019	Eyes	4	Habituation	No	*n* = 48 (21F)
Denicola et al., 2013	Face exposure	4, 8	Preferential looking	No	*n* = 64
Di Giorgio et al., 2012	Face exposure	3, 6	Visual search	Yes	*n* = 31 (17F) of which: *n* = 12 6 m (7F) *n* = 19 3 m (10F)
Doi et al., 2010	Eyes, Emotion	10	Disengagement task	Yes	*n* = 24 (13F)
Farroni et al., 2000	Gaze cueing, motion	4, 5	Eye-gaze cueing paradigm	No	Exp 1: 13 (7F) Exp 2: 16 (7F) Exp 3: 30
Farroni et al., 2002	Eyes	Birth, 4	Preferential looking	No	*n* = 17 (10F)
Farroni et al., 2005	Eyes	Birth	Preferential looking	No	*n* = 105 of which Exp 1:a: *n* = 33 Exp 1:b: *n* = 17 Exp 1:c: *n* = 12 Exp2a: *n* = 31 Exp2b: *n* = 12
Farroni et al., 2006	Eyes	Birth	Preferential looking	No	Exp 1: *n* = 15 (4F) Exp 2: *n* = 18 (8F) Exp 3: *n* = 29 (13F)
Farroni et al., 2007	Eyes	4, 5	Habituation	Yes	*n* = 24 (11F)
Fecher and Johnson, 2019	Mouth, motion	9	Switch habituation task	No	*n* = 48 (24F)
Flom and Bahrick, 2007	Emotion	3, 4, 5, 7	Habituation	No	Exp 1 (bimodal): *n* = 18 (9F) 3 m, *n* = 18 (10F) 4 m, *n* = 18 (7F) 5 m, *n* = 18 (9F) 7 m; Exp2 (auditory): *n* = 18 (11F) 4 m, *n* = 18 (9F) 5 m, *n* = 18 (8F) 7 m; Exp3 (visual): *n* = 18 (8F) 4 m, *n* = 18 (8F) 5 m, *n* = 18 (10F) 7 m; Exp 4 (asynchronous): *n* = 18 (9F) 4 m, *n* = 18 (10F) 5 m; Exp 4 (unimodal sequential): *n* = 18 (7F) 4 m.
Flom et al., 2018	Emotion	3 and 5	Habituation	No	*n* = 20 3 m *n* = 20 5 m (18F in total)
Franchak et al., 2018	Face exposure	12	Free play	Yes	*n* = 17
Fu et al., 2020	Emotion	4–24	Orienting eye-tracking task	Yes	*n* = 151 (65F)
Galati et al., 2016	Featural/configural	3, 5	Familiarization	No	Exp 1: *n* = 20 (7F) Exp2: *n* = 20 (5F) Exp3: *n* = 18 (5F) Exp4: *n* = 36 (15F)
Gamé et al., 2003	Featural/configural	2–6	Familiarization	No	*n* = 43 (22F) of which *n* = 8 (5F) 2 m *n* = 9 (6F) 3 m *n* = 9 (4F) 4 m *n* = 9 (3F) 5 m *n* = 8 (4F) 6 m
Geangu et al., 2016	Emotions	7	Familiarization	Yes	*n* = 77 UK, *n* = 76 Japan
Gliga et al., 2009	Face exposure	6	Visual search	Yes	Exp 1 *n* = 16 (8F) Exp 2 *n* = 12 (7F) Exp 3 *n* = 16 (6F)
Gluckman and Johnson, 2013	Face exposure	6	Visual search	Yes	*n* = 32 (16F)
Gredebäck et al., 2008	Gaze cuing	5–6- 9- 12-	Gaze (and head) following video with eye-tracking	Yes	*n* = 16 5 m, *n* = 16 6 m, *n* = 16 9 m, *n* = 16 12 m
Gross and Schwarzer, 2010	Emotion	7 and 9	Habituation	No	*n* = 33 (18F) 7 m, *n* = 38 (16F) 9 m
Haensel et al., 2020	Eyes, Mouth	10, 16	Free-viewing task	Yes	*n* = 48 10 m (21F) *n* = 41 16 m (16F)
Hayden et al., 2007	Featural/configural	5, 7	Movement-enhanced discrimination procedure	No	Exp 1: *n* = 24 7 m (11F) Exp 2: *n* = 32 5 m (19F)
Heck et al., 2016	Emotion	3.5 and 5	Looking time following a peripheral checkboard is presented	Yes	*n* = 24 (15F) 3.5 m, *n* = 24 (12F) 5 m
Hernik and Broesch, 2019	Gaze cuing	5–7	Gaze (and head) following videos with eye-tracking	Yes	*n* = 22 (10F)
Hillairet de Boisferon et al., 2017	Mouth	4, 6, 8, 10, 12	Free-viewing task	Yes	Exp. 1 *n* = 93 (39F) Exp. 2 *n* = 81 (39F)
Hillairet de Boisferon et al., 2018	Mouth	14 and 18	Free-viewing task	Yes	*n* = 91 (29F)
Houston-Price et al., 2006	Gaze cuing	15	Paired comparison preceded by gaze cue and auditory stimulus (online video coding)	No	Exp 1: *n* = 27 (14F), Exp 2: *n* = 30 (14F), Exp 3: *n* = 30 (18F), Exp 4: *n* = 32 (20F)
Humphreys and Johnson, 2007	Featural/configural	4, 7	Habituation	No	*n* = 32 of which *n* = 16 4 m (8F) *n* = 16 7 m (7F)
Hunnius and Geuze, 2004	Motion	6–26	Live scenes	Yes	*n* = 10 (5F) 6 to 26 months
Ichikawa et al., 2014	Motion, emotion	4–5 and 6–7	Exp 1: preferential looking static images. Exp 2: familiarization and visual preference with static images. Exp 3: habituation to static images and short videos with online video coding	No	Exp 1: 24 (9F) 4/5 m, 24 (5F) 6/7 m, Exp 2: 18 (10F) 4/5 m, 18 (12F) 6/7 m, Exp 3: 18 (5) 4/5 m, 18 (12F) 6/7 m
Ichikawa and Yamaguchi, 2014	Emotion	6–7	Habituation	No	*n* = 32 (13F)
Ichikawa et al., 2011	Motion	7–8	Preferential looking task	No	Exp 1: *n* = 29 (10F) 5/6 m, *n* = 29 (13F) 7/8 m, Exp 2: *n* = 16 (8F) 5/6 m, *n* = 16 (6F) 7/8 m
Jayaraman et al., 2015	Face exposure	1–11	Head mounted cameras, videorecording infants’ natural visual ecology	No	*n* = 22 (11F)
Jayaraman et al., 2017	Face exposure	1–24	Head mounted cameras, videorecording infants’ natural visual ecology	No	*n* = 120 (53F) of which Exp 1: *n* = 84 (34F) Exp 2: n = 36 (19F)
Kato and Konishi, 2013	Featural/configural	6–13	Visual scanning	Yes	*n* = 40 (20F) of which *n* = 10 (5F) 6 m *n* = 10 (5F) 8.5 m *n* = 10 (5F) 11 m *n* = 10 (5F) 13.5 m
Kim and Johnson, 2013	Emotion	6	Preferential looking	Yes	Exp 1: *n* = 22 (12F), Exp 2: *n* = 22 (11F)
Kim and Johnson, 2014	Motion	3, 5	Preferential looking task (eye-tracking)	Yes	*n* = 33 (16F) 3 m, *n* = 42 (21F) 5 m
Kubicek et al., 2013	Mouth, Motion	12-	Familiarization	Yes	*n* = 40 (19F)
Layton and Rochat, 2007	Motion	4–8-	Habituation/dishabituation task	No	*n* = 62
Lee et al., 2015	Emotion	6–9–12-	Habituation	No	Exp 1: *n* = 23 (9F) 6 m, *n* = 41 (21F) 9 m, *n* = 43 (21F) 12 m, Exp2: *n* = 16 (10F), *n* = 38 (18F)
Leo and Simion, 2009	Featural/configural	Newborns	Preferential looking	No	Exp 1: *n* = 14 (7F) Exp 2: *n* = 12 (4F)
Leo et al., 2018	Motion, Emotion	Newborns	Habituation (online video coding)	No	Exp 1: *n* = 28 (14F), Exp 2: *n* = 28 (14F), Exp 3: *n* = 14 (5F)
Leppanen et al., 2010	Emotions	7	Gap-Overlap	No	*n* = 42 (20F)
Lewkowicz and Hansen-Tift, 2012	Mouth	4, 6, 8, 10, 12	Free-viewing task	Yes	*n* = 179 of which Exp 1: 4 months *n* = 19 6 months *n* = 16 8 months *n* = 17 10 months *n* = 17 12 months *n* = 20 Exp 2: 4 months *n* = 19 6 months *n* = 15 8 months *n* = 17 10 months *n* = 20 12 months *n* = 19
Macchi Cassia et al., 2004	Face exposure	Newborns	Preferential looking	No	*n* = 60
Mercure et al., 2019	Mouth	4–8	Mcgurk task	Yes	*n* = 73 (34F)
Nagy, 2008	Motion	Birth	Still-face paradigm	No	*n* = 90 (42F)
Nakato et al., 2009	Gaze cuing	6, 8	Familiarization	No	*n* = 16 (6F) 6 m, *n* = 16 (7F) 7 m, *n* = 16 (7F) 8 m
Niedźwiecka and Tomalski, 2015	Eyes, gaze cuing	9, 12	Gaze cuing by different facial expressions	Yes	Pilot: *n* = 13 (5F) 9 m, Exp: *n* = 27 (13F) 9/12 m
Oakes and Ellis, 2013	Featural/configural	4.5, 6.5, 8, 12.5	Visual scanning	Yes	*n* = 24 (8F) 4.5 m, *n* = 27 (12F) 6.5 m, *n* = 21 (9F) 8 m, *n* = 20 (5F) 12.5 m
Otsuka et al., 2009	Motion	3–4	Familiarization	No	Exp 1: *n* = 24 (10F), Exp 2: *n* = 12 (5F), Exp 3: *n* = 12 (8F), Exp 4: *n* = 12 (7F)
Otsuka et al., 2016	Gaze cuing	4–5, 7–8	Wollaston’s task	No	*n* = 20 (9F) 4/5 m, *n* = 20 (7F) 7/8 m
Peltola et al., 2008	Emotions	7	Gap-Overlap	No	*n* = 17 (9F)
Peltola et al., 2009b	Emotion	7	Overlap eye-tracking task	Yes	*n* = 15
Peltola et al., 2009a	Emotions	5, 7	Visual paired comparison task	No	*n* = 23 5 m *n* = 26 7 m
Peltola et al., 2011	Emotion	7	Overlap task	No	*n* = 24
Peltola et al., 2013	Emotion	5, 7, 9, 11	Overlap task	Yes	*n* = 25 5 m, *n* = 26 7 m, *n* = 28 9 m, *n* = 25 11 m
Peltola et al., 2015	Emotions	7	Gap-Overlap	Yes	*n* = 62 (24F)
Pérez-Edgar et al., 2017	Emotion	4–24	Baby dot-probe task	Yes	*n* = 145 (63F)
Pickron et al., 2017	Eyes, Gaze cuing	5, 10	Gaze cuing + paired object comparison	Yes	*n* = 32 (21F) 5 m, *n* = 30 (18F) 10 m
Pons et al., 2015	Mouth	4, 8, 12	Free-viewing task	Yes	*n* = 60 (26F) of which *n* = 20 4 months (10F) *n* = 20 8 months (8F) *n* = 20 12 months (8F)
Pons et al., 2019	Eyes	12	Free-viewing task	Yes	*n* = 34 (20F)
Quadrelli et al., 2020	Emotion	7–8	Habituation	No	*n* = 36 (16F)
Quinn and Tanaka, 2009	Featural/configural	3–4, 6–7	Familiarization	No	*n* = 64 of which *n* = 32 3- to 4 m (15F) *n* = 32 6- to 7 m (20F)
Quinn et al., 2013	Featural/configural	3–7	Familiarization	No	*n* = 64 (36F) of which *n* = 32 (16F) 3- to 4-months *n* = 32 (20F) 6- to 7-months
Rennels and Davis, 2008	Face exposure	2, 5, 8, 11	Parent-report during two-weeks observation	No	*n* = 42 (18F)
Rhodes et al., 2002	Featural/configural	5–8	Preferential looking	No	*n* = 27 (9F)
Rigato et al., 2011a	Eyes	Birth	Habituation (1a, 1b) Preferential looking (2a, 2b)	No	Exp 1a: *n* = 16 Exp 1b: *n* = 18 Exp 2a: *n* = 8 Exp 2b: *n* = 6
Rigato et al., 2011b	Eyes, emotions	Birth	Preferential looking	No	Exp 1: *n* = 14 Exp 2: *n* = 13 Exp 3: *n* = 13 Exp 4: *n* = 16
Rigato et al., 2013	Gaze cuing	4	Modified Posner’ spatial cuing with eye gaze as cue	Yes	*n* = 14 (5F)
Rose et al., 2002	Featural/configural	7, 12	Familiarization	No	Exp 1: *n* = 72 of which *n* = 36 7 m (22F) *n* = 36 12 m (15F) Exp 2: *n* = 48 of which *n* = 24 7 m (10F) *n* = 24 12 m (14F)
Safar and Moulson, 2017	Emotion	6.5	Habituation (exp 1), preferential looking (exp 2)	No	Exp 1: *n* = 32 (23F), Exp 2: *n* = 34 (18F)
Sai, 2005	Face exposure	Birth	Preferential looking	No	Exp 1: *n* = 14 (7F) Exp 2: *n* = 14 (8F) Exp 3: *n* = 10 (5F) Exp 4: *n* = 15 (7F)
Sakuta et al., 2014	Featural/configural	3–5, 6–8	Familiarization	No	*n* = 48 of which *n* = 24 3 to 5 months (14F) *n* = 24 6 to 8 months (15F)
Schure et al., 2016	Mouth	8	Training + Habituation	Yes	*n* = 93 (44F)
Schwarzer and Jovanovic, 2010	Emotion	8	Habituation	No	Exp 1: *n* = 64 (M/F ratio missing) Exp 2: *n* = 21 (9F)
Schwarzer and Zauner, 2003	Featural/configural	8	Habituation	No	*n* = 97 (46F)
Schwarzer et al., 2007	Featural/configural	4, 6, 10	Habituation	No	Exp 1: *n* = 264 of which *n* = 88 4 m (36F) *n* = 88 6 m (39F) *n* = 88 10 m (44F) Exp 2: *n* = 75 4 m (30F)
Sebastián-Gallés et al., 2012	Mouth	8	Habituation	No	*n* = 48
Segal and Moulson, 2020b	Featural/configural, emotion	7	Preferential looking	Yes	*n* = 62 (31F)
Segal and Moulson, 2020a	Emotion	7	Free-viewing	Yes	*n* = 63 (33F)
Senju et al., 2008	Gaze cuing	9	Familiarization	No	Exp 1: *n* = 14 (6F), Exp 2: *n* = 12 (7F), Exp 3: *n* = 12 (7F), Exp 4: *n* = 12 (8F)
Senju et al., 2008	Gaze cueing	6.5	Gaze (and head) following	Yes	Exp 1: 20 (10F), Exp 2: 20 (10F)
Senju et al., 2015	Gaze cueing	6–10, 12–16	Gaze (and head) following	Yes	*n* = 14 (7F)
Simpson et al., 2014	Featural/configural	4–6, 9–12	Familiarization	No	*n* = 77 (34 F) 4 to 6 m *n* = 66 (27 F) 9 to 12 m
Simpson et al., 2020	Face exposure	2, 4, 6	Visual search	Yes	*n* = 65
Souter et al., 2020	Mouth	18–30	Free-viewing	Yes	*n* = 58
Spencer et al., 2006	Motion	3–8	Familiarization	No	*n* = 55 (26F)
Streri et al., 2013	Mouth, Motion	Birth	Familiarization	No	not specified
Streri et al., 2016	Mouth	3–9	Familiarization	No	*n* = 72 of which *n* = 24 3 m (11F) *n* = 24 6 m (12F) *n* = 24 9 m (9F)
Striano et al., 2007	Gaze cuing	1.5, 3	Live interaction	No	Exp 1: *n* = 12 (9F) 6-wo, *n* = 14 (8F) 3 m, Exp 2: *n* = 22 (10F) 6-wo
Sugden and Moulson, 2019	Face exposure	3-	Head mounted cameras, videorecording infants’ natural visual ecology	No	*n* = 40 infants (13F) 169 h, 58 min, and 8 s of video recorded
Szufnarowska et al., 2014	Gaze cueing	6	Gaze (and head) following	Yes	*n* = 20 (14F)
Tenenbaum et al., 2013	Mouth	6, 9, 12	Free-viewing task	Yes	*n* = 97 at 6 months (44F) *n* = 65 at 9 months (29F) *n* = 62 at 12 months (29F)
Thompson et al., 2001	Featural/configural	7	Preferential looking	No	Exp 1: *n* = 39 (22F) Exp 2: *n* = 18 (9F)
Tomalski et al., 2013	Mouth	6, 9	Free-viewing task	Yes	*n* = 32 (22F)
Tsurumi et al., 2019	Face exposure	5–8	Preferential looking	No	*n* = 20 (7F)
Turati and Simion, 2002	Featural/configural, face exposure	Newborns	Exp. 1: habituation exp. 2: habituation exp. 3: familiarization exp. 4: habituation	No	Exp. 1: *n* = 58 (28F) Exp. 2: *n* = 59 (31F) Exp. 3: *n* = 25 (14F) Exp. 4: *n* = 26 (12F)
Turati et al., 2004	Featural/configural	4	Habituation	No	Exp 1: *n* = 14 (7F) Exp 2: *n* = 33 (14F)
Turati et al., 2005	Face exposure	Birth, 3	Preferential looking	Yes	Exp 1: *n* = 16 (8F) Exp 2 *n* = 34 Exp 3 *n* = 10
Turati et al., 2008	Featural/configural	Newborns	Habituation	No	Exp. 1: *n* = 18 Exp. 2: *n* = 18 Exp. 3: *n* = 18 Exp. 4: *n* = 17
Turati et al., 2011	Emotion	3	Familiarization	No	Exp 1: *n* = 73 (39F), Exp 2: *n* = 22 (11F)
Valenza et al., 2015	Motion	Birth and 4	Gap-Overlap	Yes	Exp 1a: *n* = 20 (6F) 4 m, Exp 1b: *n* = 14 newborns, Exp 2a: *n* = 18 (9F) 4 m, Exp 2b: 14 newborns
Von Hofsten et al., 2005	Gaze cuing	12	Video of gaze (and head) following and pointing	Yes	*n* = 20 (8F)
Wagner et al., 2013	Eyes, Mouth	6, 9, 12	Preferential looking	Yes	*n* = 36 6 m (15F) *n* = 42 9 m (18F) *n* = 39 12 m (24F)
Walle and Campos, 2014	Emotion	16, 19	Video coding infant behavior	No	*n* = 35 (20F) 16 m, *n* = 30 (14F) 19 m; *n* = 38 (20F) 16 m, *n* = 41 (24F) 19 m
Xiao and Emberson, 2019	Emotion	9	Preferential looking between two images following auditory presentation (eye-tracking)	Yes	*n* = 18 (9F)
Xiao et al., 2015	Motion	3, 6, 9	Familiarization	Yes	*n* = 41 (17F) 3 m, *n* = 32 (16F) 6 m, *n* = 26 (12F) 9 m
Yamashita et al., 2012	Eyes	6, 8	Familiarization	No	*n* = 24 (12F)
Yong and Ruffman, 2016	Emotion	7	Matching to sample task (face image and auditory stimulus expressing emotion)	No	*n* = 24 (10F)

### Environmental Exposure to Faces

Early in life, infants often spend most of their time inside the household. As they grow, their living contexts extend and they encounter more people outside the family (i.e., peers and teachers). To get a sense of the likelihood of being exposed to masked faces in daily routines, we summarize naturalistic and screen-based studies on the extent to which faces are present and looked at in everyday visual environments during the first 3 years of life.

Studies conducted using head mounted cameras showed that within the first year, the amount of face exposure is higher for younger infants; infants see fewer faces as they grow older during the first 2 years of life (Jayaraman et al., 2015, 2017). Indeed, 3-month-old infants are exposed to faces for 21% of their daily time, and this is most frequently the face of the primary caregiver. However, frequency of exposure and consistency of faces vary across contexts, with caregiver’s faces being present in a wide range of contexts but for shorter durations compared to other relatives or strangers (Sugden and Moulson, 2019). Similarly, in a survey study, Rennels and Davis (2008) found that over the first year of life, most adult-infant interactions happen with the primary caregiver and with people of the same age, gender, and race. Furthermore, female faces appear more frequently in infants’ visual environment; infants have 2.5 times more experience of the mother’s compared to the father’s face (Rennels and Davis, 2008). In terms of duration, mean exposure time to unfamiliar individuals shortens with age, possibly because infants move around the environment and shift attention away from faces more frequently as they grow (Rennels and Davis, 2008). At 12 months of age, when infants’ motor abilities are rapidly developing and performing actions might require some effort, face looking and mutual gaze are decreased when parents are standing and face looking has higher motor costs (vs. a low motor cost condition), as shown by eye-tracking data collected during free play. Indeed, parents are keen on spending time on the floor, perhaps to facilitate face looking in their children (Franchak et al., 2018).

Differently from naturalistic studies, screed-based studies show that with age, infants look more at faces when exposed to complex and dynamic social contexts. Within complex arrays, faces attract and hold infants’ attention as in adults at 6 but not 3 months of age (Di Giorgio et al., 2012) and are looked at for longer than objects (Gluckman and Johnson, 2013) or toys at both 4 and 8 months (DeNicola et al., 2013). Orienting to faces is facilitated by direct gaze before 6 months (Simpson et al., 2020), in line with literature supporting the role of direct gaze in engaging attention from the earliest developmental stages (Farroni et al., 2002). After 6 months of age, infants pay increasing attention to moving faces, compared with static images of patterns (Courage et al., 2006). At this age, both upright and inverted faces elicit attention orienting in complex visual displays, but only upright faces hold infants’ attention (Gliga et al., 2009).

Taken together, this evidence suggests that a significant amount of time is spent looking at faces from early in life. While an increase in face looking with age is found when presenting infants with complex arrays in laboratory settings, naturalistic studies highlight that infants look less at faces as they grow. The motor skills required to direct attention to faces in real life situations, as well as the increasing importance of the adults’ hands and objects in social contexts could perhaps explain some of these contrasting results. In lab settings, when face exposure does not depend on postural motor skills, infants increasingly find images of faces more engaging than objects, especially if presented upright and with direct gaze. Thus, they gradually show a preference for the stimuli they are largely exposed to that will scaffold their face perception and social communication skills.

### The Development of Face Perception

Faces are a predominant stimulus in an infant’s environment and constitute an important source of learning from soon after birth. Wearing a face mask changes low-level perceptual properties of faces that include contrasts (involving borders and features) as well as the features that are visible. Knowledge of the mechanisms that underpin face perception from birth is necessary to understand whether and when face coverings could impact face perception.

Newborns are predisposed to orient toward face-like configurations (Morton and Johnson, 1991; Gamé et al., 2003; Macchi Cassia et al., 2004) and multiple studies have been conducted over the years aiming to explain mechanisms beneath face preference at birth.

One proposed mechanism is that stimuli with more elements on the upper part—two eyes vs. one mouth in faces—are preferred due to the presence of more receptors, and consequently higher sensitivity, in the part of the retina that perceives the upper visual field (top-heavy hypothesis; Simion et al., 2002). Supporting this hypothesis, Macchi Cassia et al. (2004) found that newborns preferred stimuli with more elements in the upper part regardless of them being a face and concluded that a non-face-specific perceptual bias could account for face preference at birth. At 3 months, when infants’ looking behavior start to be less influenced by automatic processes and they can discriminate top- vs. bottom-heavy stimuli (Chien et al., 2010), Turati et al. (2005) and Chien (2011) found no consistent bias for top-heavy patterns.

Another proposed mechanism for face bias could be linked to low-level visual constraints, as newborns’ looking behavior is strongly affected by low-level stimulus properties, such as image contrast, and their vision is tuned to low frequencies (black and white changes). Relatedly, a primitive subcortical mechanism (CONSPEC, Table 3) could support face detection processes at birth, being later complemented by a domain-relevant mechanism (CONLERN) that gradually enables the system to recognize the face *per se* instead of a general face-like configuration (Morton and Johnson, 1991; Johnson, 2005; Johnson et al., 2015). Supporting this account, de Heering et al. (2008) manipulated the spatial frequencies of faces to which newborns were habituated and found that face recognition is facilitated by the lowest spatial frequency within the visible range. Further studies manipulating phase contrast of the stimuli revealed that face-characteristic contrast polarity (one or more dark areas surrounded by lighter surface) is required for the upright face preference in newborns (Farroni et al., 2005). The importance of contrasting internal features of faces for face preference was also found in older infants. By 3 months, infants looked longer at face than car images when faces were manipulated using a horizontal filter that altered external borders and the nose feature but preserved the face configuration composed by eyes and mouth. No face preference was shown when images were manipulated with a vertical filter, preserving the face shape but altering the top-heavy face pattern, and with inverted faces (de Heering et al., 2015).

**Table 3:** Glossary.

Configural processing	Holistic way of processing whereby features are integrated into a Gestalt to extract meanings (Kimchi, 1992). Expertise in face processing is based on the ability to encode configural information, useful to extract communicative meanings conveyed by emotional expressions, gaze cueing and identities. As all faces share the same general configuration (e.g., eyes are above the nose, called first-order relations), to distinguish a face from another we need to rely on more subtle changes of spatial relation among features (e.g., the distance between the eyes, named second-order relations) (Carey and Diamond, 1977)
CONSPEC	A primitive subcortical mechanism that could support face detection processes at birth, later complemented by a domain-relevant mechanism (CONLERN) that gradually enables the system to recognize the face *per se* instead of a general face-like configuration (Morton and Johnson, 1991; Johnson, 2005; Johnson et al., 2015).
Fearful attentional bias	Enhanced attention to fearful faces compared to other emotional or neutral faces
Featural processing	Detailed-oriented style where features are processed independently from their context (Kimchi, 1992)
Intersensory redundancy	Used referring to information coming from the mouth, implies the presence of synchronous visual and auditory cues (Lewkowicz and Hansen-Tift, 2012)
Inversion effect	Integrating single facial features into a configuration is easier when the facial stimulus is upright than when the face is inverted (Yin, 1969)
McGurk effect	Based on the McGurk effect, the task consists in the presentation of faces articulating syllables with congruent, incongruent and silent auditory tracks.
Wollaston illusion	This illusion postulates that eyes orientation is evicted based on the direction of the face too

Hypotheses on the implications of mask wearing on face preference in early life might differ according to the aforementioned theories. Referring to the top-heavy theory, newborns’ exposure to masked faces in the first weeks of life (for example, in case of prolonged hospitalization after birth) should not inhibit face bias as the presence of more elements in the upper part of the stimulus is maintained. However, since this theory is based on the interdependence between the stimulus borders and the internal features (Turati and Simion, 2002), one question remains on whether masked faces are perceived as oval shapes or whether the upper border of the mask is perceived as a face bound. In the latter case, the stimulus composed by forehead and eyes (face region above the mask) would not show the top-down asymmetry and face bias could possibly be inhibited. In the CONSPEC-CONLERN framework, preferential orienting to masked faces at birth supported by subcortical neural pathways (CONSPEC) is expected to be maintained, as contrasts are preserved in the eyes region. One could wonder whether, if infants are exclusively exposed to masked faces in the first 2 months of life, the CONLERN system might theoretically be disadvantaged as it would receive atypical input regarding the face configuration. However, a recent update of the two-process theory of face processing highlights the central role of eye contact in subcortical rapid face detection (Farroni et al., 2002; Johnson et al., 2015). Since eyes are not impacted by face covering, this may compensate for the missed exposure to the entire face configuration for the development of cortical pathways underlying CONLERN. Interestingly, Sai (2005) found that head turns toward the mothers’ face occur only in the presence of mothers’ voice, suggesting that auditory stimuli also contribute to the origins of face processing and might support face preference when the stimulus is partially occluded by the mask; however, this hypothesis has not been tested yet. Besides, some of this will depend on whether there is a critical period, and how long that extends for, given that newborns would presumably be more exposed to masked faces when in the hospital while once at home they would probably see unmasked faces.

#### Featural and Configural Face Processing

When looking at a face, two different perceptual strategies can be adopted to encode information: featural and configural (Table 3). Expertise in face processing is based on the ability to encode configural information, useful to extract communicative meanings conveyed by emotional expressions, gaze cueing, and identities. Disruptions in the presentation of the typical face configuration have been shown to affect configural processing (i.e., Inversion Effect, Table 3). This is also the case of face masks, as shown in adults (Freud et al., 2020). Whether and to what extent face masks have similar effects in developmental populations is currently unreported. To generate hypotheses on the potential effects of mask wearing on featural and configural face processing from early in life, in this paragraph, we summarize evidence on the emergence and development of these scanning strategies in infancy.

Developmental changes in the strategies employed to encode facial information have been investigated to explore the pathways leading to specialized face processing. Configural face processing appears to gradually develop during the first year of life (Thompson et al., 2001; Bhatt et al., 2005). At birth, newborns’ ability to discriminate face-like patterns relies on their inner features (Turati and Simion, 2002), although there is evidence that they do not need to rely on fine details and spatial relation between features to recognize face-like patterns (Leo and Simion, 2009). A perceptual shift from featural to configural processing is suggested to happen between 4 and 10 months (Schwarzer et al., 2007), with configural face sensitivity to fine spatial resolution specializing sometime between 3 and 5 months of age (Bhatt et al., 2005). For example, Quinn and Tanaka (2009) found 3-month-olds to be more sensitive to configural changes (distance variations between features) than local changes (variations in features’ size) around both the upper (eyes) and lower (mouth) face areas. Between 3 and 7 months, they appear to specialize in detecting local changes happening in the upper vs. lower face region (Quinn and Tanaka, 2009). However, the same effect has been found with objects, suggesting that processing of featural and configural variations might not be face-specific (Quinn and Tanaka, 2009; Quinn et al., 2013). Differently, despite sensitivity to featural and first-order changes being present as early as 3 months, sensitivity to variations in spatial distance among features could only be observed in 5-month-olds (Bhatt et al., 2005). During the second half of the first year of life, infants scan upright faces more efficiently (Kato and Konishi, 2013; Simpson et al., 2014) and, like adults, at 7–8 months they are faster in identifying upright than inverted faces (Tsurumi et al., 2019). While scanning patterns of the different face regions (high, middle, and low) are comparable for upright and inverted faces before 8 months, infants gradually start to scan upright faces more broadly and do so significantly more than inverted faces by 1 year of life (Oakes and Ellis, 2013). Thus, the inversion effect strengthens during the first year, possibly due to infants’ experience with the entire face configuration. The end of the first year seems to be a crucial period for integrating features within the typical upright face configuration, and sufficient exposure to the entire face could be important.

Infants’ face processing ability varies according to different factors beyond age, such as face orientation and pose. The ability to recognize (i.e., show novelty preference following habituation) unfamiliar full faces presented on a ¾ pose is recorded as early as 1–3 days of life (Turati et al., 2008). At 4 months, infants’ performance in face recognition takes advantage of the face being upright if they had been familiarized with different poses of the same face, indicating that this manipulation requires more cognitive resources for face recognition (Turati et al., 2004). At the same age, but not at birth, infants are faster to orient from a central face toward a peripheral face when this is upright than inverted, although motion of the central face stimulus (displaying blinking, mouth opening, or nodding) reduces the speed of orienting toward upright and inverted faces (Valenza et al., 2015). While some studies indicate sensitivity to face orientation at birth (Leo and Simion, 2009), others indicate that from 4 months infants’ face processing ability is sensitive to factors like orientation, pose, and motion that modify the entire face configuration. As argued by Turati et al. (2004), differential sensitivity to inversion indicates a progressive tuning to the characteristics and configuration of a face. Since face masks affect the visible face configuration, it is possible that speed of detection and recognition of masked faces could be altered from 4 months of age.

The number of full unfamiliar faces a child is exposed to can be a factor that affects face expertise, since exposure to multiple different faces provides more opportunities to explore second-order relations. While between 3 and 4 months of age infants do not spontaneously detect changes in spacing among facial features, they can be trained to do so by being repeatedly exposed to faces varying in spatial proportions (Galati et al., 2016), in line with the idea that this is a critical period for developing configural face processing skills. On the contrary, 5- to 8-month-old infants spontaneously use configural face processing as they demonstrate sensitivity to variations in spatial relations among face features that are within the normal range of human variability (Hayden et al., 2007). When presented with pairs of faces where location of spatial features was manipulated, 5- to 8-month-old infants demonstrate sensitivity to symmetry and averageness, reflected by increased looking toward less average/symmetric faces (Rhodes et al., 2002). Accordingly, 7-month-olds look less at shortened and elongated faces, where distance between features are atypical, than faces with an average eye-to-mouth distance (Thompson et al., 2001). Furthermore, Humphreys and Johnson (2007) habituated 4- and 7-months-old infants to morphed faces and found that face regions used for identity recognition narrow with age, allowing more refined recognition and less errors with increased experience of faces (Humphreys and Johnson, 2007). The variety of faces infants are exposed to is important to develop and refine face processing skills. If mask wearing is mandatory outside the home environment and infants only see caregivers without masks, it is possible that identity recognition skills are affected.

Supporting the importance of experience with faces in everyday contexts is the research by Cashon et al. (2013). They found that sitting abilities correlate with configural face processing in 6-month-olds, suggesting that the development of more mature face processing systems based on configural instead of featural strategies also depends on changing in viewpoint and context linked with motor skills. Further, 7-month-old infants can confidently use configural information to discriminate upright but not inverted faces (which are likely never seen in the normal environment; Cohen and Cashon, 2001). Moreover, Schwarzer and Zauner (2003) showed that configural processing is used by 8 month olds to encode facial information coming from real human faces, while featural processing takes place when presented with face-like configurations (handmade drawings). Configural strategies are increasingly employed from 6 to 12 months, but older infants also use featural information for face processing (Rose et al., 2002). Moreover, Sakuta et al. (2014) showed that from 6 months onward infants can discriminate faces according to eye size, while 3 to 5 month olds could not. This suggests that building expertise on eyes alone could compensate for the diminished expertise on full face configuration in face recognition tasks in case of preponderant exposure to masked faces.

Taken together, these studies describe a gradual transition from featural to configural processing with infants using different strategies according to their developmental stage as well as experience with face configurations. Specifically, at birth, newborns rely on internal features to discriminate between faces or face-like patterns. The literature overall supports a transition to using configural strategies for face recognition between 3 and 5 months of age. Configural face processing abilities are clearly manifested from 7 to 8 months and are increasingly used for face recognition toward the end of the first year for upright faces. The development of configural processing is likely driven by experience, possibly with a range of faces. Thus, if infants are just seeing a very small number of people unmasked, it is possible that these skills will develop differently. Featural strategies are used when the configuration is broken, as it happens in the case of inverted faces. They might therefore be used for recognition of masked faces too. Furthermore, we know that infants pay different attention to eyes and mouth according to their developmental needs (i.e., attentional shift to the mouth for language learning; see paragraph 3.2.2). While masks drastically change visibility of facial features, it is possible that they impact infant’s perception of the face configuration differently at different ages. Although their presence could break the CONSPEC (Acerra et al., 2002), this might not affect the communicative valence of the face at birth and throughout the first few months of life, when eyes are more salient than the mouth, while this could happen when attentional shifts to the mouth occur. However, it is also possible that being exposed to a more limited number of faces in a variety of situations (i.e., different distance, lighting, orientation, and expression) could be enough to support the development of configural strategies. Whether masked faces disrupt facial information processing and whether this effect is age-specific remain open questions for future research.

### Perceiving Facial Features and Their Communicative Meanings

The development of face processing abilities partly relies on infants’ attention being focused on different facial features during sensitive periods for the development of functions and skills. Crucially, faces are one of the most prominent sources of social communication. Perceptual information from the face contributes to shape trajectories of individual socio-communicative skills. For instance, eye contact engages infants (Farroni et al., 2007) and gaze shifts support their attention allocation in the environment to learn from relevant stimuli (see, for example, Cetincelik et al., 2021), while information coming from others’ mouth supports language development (Lewkowicz and Hansen-Tift, 2012). Face masks change what features can be perceived, covering nose and mouth while leaving the eye region and forehead uncovered. To discuss whether and when face masks could interfere with infants’ socio-cognitive development, we examine published studies on infants’ focus on each facial feature and on how social and communicative skills are learnt from others’ faces.

#### The Value of Interactive Faces

Soon after birth, newborns appear to preferentially orient to stimuli that carry a socio-communicative meaning, which are preferred over non-communicative cues. Differently to when they are habituated to still faces, newborns do not show novelty preference after being habituated to a live interaction scene where they saw a face producing communicative cues (Cecchini et al., 2011). In classic habituation studies, novelty preference is interpreted as evidence that children can discriminate between the stimulus they have been habituated to and the one they see for the first time. Thus, these results could be interpreted as in favor of the motionless vs. interactive face. On the contrary, the authors argue this proves that newborns’ interest is enhanced and more durable for interactive faces, and therefore, they are equally attracted to both post-habituation faces, regardless of previous exposure. In line with this view, newborns show a significant decrease in the looking time to faces that are not responsive during social interaction versus interactive faces (Nagy, 2008). Moreover, Coulon et al. (2011) and Streri et al. (2013) found that newborns look longer at faces when they have previously been familiarized to a video of the same face with a direct gaze, interacting or talking to them. They also seem to be facilitated in identity recognition when familiarized with dynamic (but not static) emotional faces, as shown by Leo et al. (2018). From these results, it is evident that newborns are wired for interactions; within those, they detect and prefer elements that build up the basis for social communication.

Interactive faces seem to be more powerful than static, non-interactive faces in attracting attentional resources and facilitating the acquisition of face processing skills across the first year of life. Kim and Johnson (2014) found that both 3- and 5-month-old infants look longer at faces directed toward them and in the presence of infant-directed speech. Moreover, faces displaying changes in facial expression facilitate face recognition in 3- to 4-month-olds (Otsuka et al., 2009) and around 5 months, infants can recognize actors based on their actions if exposed for enough time (min 320 s) to the naturalistic scene (Bahrick et al., 2002; Bahrick and Newell, 2008). Similarly, Spencer et al. (2006) showed that infants aged between 3 and 8 months can discriminate between people based on differences in their facial motion. Layton and Rochat (2007) tested whether motion or visual contrast helped infants discriminate their mother from a stranger to which they had been habituated. They found that facial motion improved recognition in 8- but not 4-month-old infants, indicating that dynamic changes are not only encoded but also used for identity recognition by 8 months of age. Of note, when using animated face patterns instead of real faces, infants preferred to look at biologically plausible vertical movements of the internal features (simulating eyes and mouth closure) compared to horizontally moving patterns only at 7–8 months of age and not at 5–6 months (Ichikawa et al., 2011). By obscuring mouth dynamics, masks partly reduce the availability of communicative cues in a face while they leave eye information only available. This could possibly influence face preference or depth of processing. We examine below what information infants receive from the different features to understand whether and when their role is essential for socio-cognitive development.

#### Eyes

##### Perception

Perceiving eyes scaffolds the development of face processing from birth. Farroni et al. (2005) conducted a series of experiments manipulating contrast within face-like patterns and real face stimuli. Results showed that newborns’ basic visual capacity is sufficient to perceive eyes within a face, and the authors suggest that this might be a reason for face preference to be manifested soon after birth. Perceiving differences in eyes direction is important not only for face detection, as discussed earlier, but also for identity recognition. Newborns can recognize a previously seen face and this process is facilitated by direct gaze (Rigato et al., 2011a). Averted gaze prevents newborns to display a preference for happy facial expression, that is, conversely observed in the presence of direct gaze (Rigato et al., 2011b). Similarly, Farroni et al. (2007) showed that 4-month-old infants manifested a novelty preference when the face they were previously habituated to had direct but not averted gaze. In older infants, eye contact has been shown to facilitate facial discrimination as well, possibly affecting three-dimensional face recognition. In fact, 8-month-old infants were able to recognize a face they were previously familiarized with even if this was rotated, but only if the familiarized face had a direct gaze (Yamashita et al., 2012).

Perceiving gaze shifts is also a crucial feature that contributes to the emergence of processing skills, as infants learn to extrapolate information about the context from the direction of eye gaze. At 4 months, infants can already orient in the direction cued by the gaze and perform shorter saccades to a peripheral object appearing in the direction of the eye gaze of a central face image (Farroni et al., 2000). Of note, this eye-gaze effect is canceled out if faces display emotional expressions, as these seem to hold their attention and reduce speed to orient toward the referent object (Rigato et al., 2013).

The ability to discriminate eye-gaze direction is at the base of another face processing skill that emerges very early in life, that is, the ability to integrate information about the head and eyes orientation when interpreting directional cues. Otsuka et al. (2016) used artificially created realistic face images in a paradigm inspired by the Wollaston’s effect (Table 3). They found that infants could infer the direction of the gaze based on the head orientation from 4 to 5 months of age. Nakato et al. (2009) investigated the same effect familiarizing infants to the original Wollaston’s drawings and saw that 8-, but not 6- and 7-month-olds, looked longer at illusory direct gaze, providing evidence that they were sensitive to the Wollaston’s effect. Inverted faces disrupt configural processing and inhibit the interpretation of gaze direction in the context of head orientation in the younger infants (Nakato et al., 2009). Thus, while at 4 months of age infants use eye-gaze direction to choose where to direct their attention, the ability to integrate information about eye gaze and head orientation especially in realistic situations develops more gradually until 8 months.

As perceiving the eyes plays a specific role in face processing from birth and the facilitatory role of eye contact and gaze shifts is preserved in more complex tasks as infants grow older, it is reassuring that the eyes region is not covered as a precaution against COVID-19 diffusion. Relatedly, examining studies investigating the role of eyes for developing socio-communicative skills is crucial for the scope of this review.

##### Communication

Within the face, eyes are a central component for communication. It is not just the quantity of faces infants are exposed to that affects the development of social brain networks—whether faces include eyes looking toward or away from the observer is crucial. Gaze direction can provide two types of social information: eye contact establishes a communicative context between humans, gaze shifts can also be interpreted as initiating “joint attention.”

Eye contact is involved in face detection processes soon after birth. Newborns not only manifest a preference for faces and face-like configurations, as discussed, but among faces they prefer those with direct eye gaze. Farroni et al. (2002) presented 2- to 5-day-old newborns with pairs of faces manipulating the direction of the gaze while keeping the face identity constant and found more frequent orientations and longer looking times toward faces with direct rather than averted gaze. In a subsequent study, they crucially found that the effect is present with upright and straight-ahead faces only (Farroni et al., 2006), that is, in the typical presentation of a face during interaction. Direct gaze also facilitates face recognition in 4-month-old infants (Farroni et al., 2007). Supporting the view that infants are tuned to detect communicative meaningful stimuli contributing to their social development, infants who looked more to their mothers’ eyes at 6 months as well as those who paid greater attention to the talker’s eyes (vs. mouth) at 12 months were found to manifest higher social and communication skills at later ages (Wagner et al., 2013; Pons et al., 2019). Attention to the eyes at these preliminary stages allows infants to engage with and learn from eyes, which support socio-cognitive development and could compensate the effects of mask wearing at later developmental stages.

The direction of the eyes constitutes an important modulator of face processing since early in life, which is integrated with multiple sources of social information. For example, eye gaze modulates infants’ allocation of attention toward emotional expression. Doi et al. (2010) found that at 10 months, infants are faster to orient toward the peripheral target in case of a central happy face with direct gaze, while it takes them longer to disengage from the central facial stimulus when the face displays anger (both if direct and averted gaze). Nevertheless, recent evidence shows that when provided with alternative communicative sensory stimulation (i.e., affective touch) infants still engage with less or non-communicative faces, suggesting that different senses conveying communicative information might compensate for each other. For example, evidence shows that when habituated to faces with averted gaze while simultaneously caressed, 4-month-olds discriminate and recognize the familiar face despite gaze being averted (Della Longa et al., 2019). This is in line with the idea that multiple sensory channels support infants’ face processing and learning. For the scope of this review, this is encouraging as it suggests that communicative meanings might enter the system through different sensory gateways and do not rely exclusively on the visual information available from a face when this is limited by mask wearing.

By the end of the first year of life, infants appear to understand the referential essence of gaze that allows to establish joint attention (Mundy, 2018). Many have studied when and how this mechanism develops. Striano et al. (2007) showed that infants start to gaze more in the direction cued by the adults’ gaze from 6 weeks to 3 months of age. While the degree to which infants looked at the experimenter during live joint attention situations did not differ by age, 3 month olds looked more at the gazed-at object compared to younger infants (Striano et al., 2007). Gredebäck et al. (2008) found that when watching an adult gazing and turning the head toward one of two possible toys, infants aged 5 to 12 months looked significantly more at the attended toy, with no effect of age on overall looking time. The microstructure of the infant gaze revealed that 5-month-olds were equally likely to perform the first gaze shift toward the attended and the unattended toy. Differently, 6-, 9-, and 12-month-old infants oriented their gaze toward the toy immediately. These findings indicate that the ability to orient the gaze following a gaze cue is not fully developed at 5 months of age.

Other information usually provided in conjunction with gaze shifts facilitates infants in processing gaze cues in the first year of life, including head direction, familiarity with the person performing the eye-gaze shift, and ostensive communicative signals. For example, at 3 to 4 months of age, head turns in the adult encourage infants to look in the direction of the adult’s moving hands and objects (Amano et al., 2004). At 5 and 10 months of age, infants seem to rely more on gaze cueing coming from highly familiar (i.e., of the race and sex infants were more exposed to) compared to non-familiar adult models (Pickron et al., 2017). Thus, it is possible that as early as 5 months of age, infants have already learnt the referential value of eye gaze coming from the caregivers. At 6 to 9 months of age, infants orient toward the cued toy first and more frequently in the presence of ostensive communicative cues, such as direct gaze and eyebrows lift or infant-directed speech preceding gaze following (Senju and Csibra, 2008; Senju et al., 2008). This is also observed in non-communicative attention-grabbing situations (e.g., if the model actor performed a shiver before the gaze shift) suggesting that attention, rather than communicative intent, plays a crucial role in eliciting gaze following (Szufnarowska et al., 2014). Perhaps in contrast with this account, a study with infants living in a rural society island in Vanuatu, where face-to-face interactions between infants and adults are less common than in Western cultures, confirmed that orienting toward the other’s gaze direction is not dependent on cultural aspects, but rather on the communicative engagement with the infant before gaze cueing. Vanuatu infants between 5 and 7 months oriented toward the cued object more easily after being addressed with infant-directed speech, compared to adult-directed speech, just like Western infants (Hernik and Broesch, 2019). Thus, the roots of joint attention seem to rely on gaze cuing from 3 to 9 months of age and are boosted, in this age range, by additional information that are not impacted by mask wearing, such as the head direction, familiarity, direct gaze, speech, and head movements.

Toward the end of the first year of age, infants start to integrate gaze direction with other communicative cues, such as facial emotional expression, pointing, and gestures, although eyes remain the most salient source of information until 2 years of age. Using a gaze-cueing task whereby faces manifested emotional expressions (happy, fearful, and angry), Niedźwiecka and Tomalski (2015) found that infants aged between 9 and 12 months were faster in orienting toward a peripheral stimulus in trials where the central stimulus was a happy face with gaze directed toward the same side of the screen. Of note, this gaze cueing effect was present only with happy facial expressions, confirming infants’ tendency to rely more on gaze information provided by positive-valanced faces. By 1 year of age, eye gaze or a combination of eye gaze and pointing, but not pointing alone, toward an object facilitates infants’ gaze shift toward the cued object, showing that gaze is still the preferred cue for learning about the surrounding environment (Von Hofsten et al., 2005). Further, during the second year of life (14 and 18 months), infants are more inclined to look in the direction cued by the adults’ eyes rather than head alone, as observed during a live gaze following task (Brooks and Meltzoff, 2002). However, typically developing children start to direct their attention more toward the adults’ hands for learning and communication from the second year of life. Chen et al. (2020) analyzed joint attention episodes during free play between parent and children using head mounted eye-trackers in children with hearing loss and children with normal hearing matched for chronological (24 to 37 months) and hearing age (12–25 months). They found that from the second year of life, hearing children tend to attend more to the parents’ hand actions, while children with hearing loss rely still more on the parents’ eye-gaze cuing (Chen et al., 2020). Eyes seem to be such a powerful communicative cue that they probably partly compensate for the absence of language information in toddlers with hearing loss.

Eye-gaze cueing is even supporting the development of language skills in the second year of life. For example, when watching a short video of a woman directing her gaze and head toward one of the two objects, 15-month-old infants looked longer at the image corresponding to the word sound played in the test phase (Houston-Price et al., 2006). This indicates that eye-gaze cueing facilitates learning of new words and is promising regarding the possibility that eyes support language acquisition even more importantly when the visual information of the mouth is less available due to mask wearing of the speaking adult. Masked faces probably convey lots of social communicative information through the eyes, so communication is likely to be less affected by masks. Effects might be observed in developmental processes that require mouth input.

#### Mouth

##### Perception

Redundant audiovisual information (see glossary on Table 3) is important for speech learning especially during the second half of the first year of life, when infants start to shift their attention from the eyes toward the mouth region, while it creates competition between attentional resources before 3 months (Bahrick et al., 2013). At 3 and 6 months, visual scanning between moving and static faces does not differ, while at 9 months infants shift their fixation more frequently between inner facial and look more at the mouth (vs. eye) region only when familiarized with dynamic faces (Xiao et al., 2015). These results are in line with findings by Oakes and Ellis (2013) with static upright face, which indicated that 4.5- and 6.5-month-old infants look more at eyes and 12-month-olds look more at the mouth. A similar pattern was found by Hunnius and Geuze (2004) who followed up 10 infants longitudinally from 6 to 26 weeks when looking at the mothers’ face. These results could be explained by the increasing importance of mouth looking for speech learning. In fact, from 8 to 10 months, infants’ attention to non-speaking faces is distributed across eye and nose regions (Liu et al., 2011; Wheeler et al., 2011; Geangu et al., 2016) while if the faces are accompanied by speech the moving mouth becomes more salient than the eyes (Lewkowicz and Hansen-Tift, 2012; Haensel et al., 2020). Consistently, a longitudinal study by Tenenbaum et al. (2013) showed that infants shifted their attention to the mouth in the presence of spoken language, but not in the presence of a smile with no language. This effect was observed from 6 to 12 months, with a significant increase between 6 and 9 months of age, concomitantly to the canonical babbling stage. Crucial for the aim of this review to evaluate effects of masks covering the mouth regions, the authors noticed high variability between subjects, suggesting that individual experience interacts with developmental needs to influence how infants deploy their attention over talking faces (Tenenbaum et al., 2013).

It is possible that mouth looking has a key role in the initial phases of speech learning. Lewkowicz and Hansen-Tift (2012) found that while looking at speaking faces (either using infants’ native and non-native language) infants focus more on the mouth from 8 months but they shift their attention to the eye region at 12 months in the native language condition only. At this age, infants have gained experience in their native language and audiovisual information is no longer useful, while they continue to attend to speakers’ mouths in the non-native language condition. The authors suggested that to gain expertise with their native language, infants need to rely on redundant audiovisual information, as they learn how to articulate speech-like syllables by imitating the talkers’ mouth (Lewkowicz and Hansen-Tift, 2012). Similarly, Schure et al. (2016) noted that despite the growing expertise in their native language, at 8 months, infants are interested in information coming from the mouth when it includes non-native speech sounds that contrast with native vowel categories they already know. At 9 months, increased looking to the mouth is observed when infants are presented with incongruent audiovisual information (e.g., seeing a mouth articulating a sound while listening to another; Tomalski et al., 2013), while at 12 months, infants focus on the mouth if they hear non-native language (Kubicek et al., 2013). Also supporting experience dependency of face processing, Fecher and Johnson (2019) found that after habituation with a face paired with a voice, 9-month-olds bilinguals subsequently looked longer to faces paired with a different versus the same voice. Thus, it seems that mouth looking plays a significant role in language learning at multiple development stages, both when speech is novel to infants and when they are in the process of learning it. Indeed, Hillairet de Boisferon et al. (2018) suggested that a second attentional shift toward the mouth region might occur when entering the word acquisition phase of language development, regardless of the spoken language being the child’s mother tongue or not. They showed that 14- and 18-month-olds monolingual English infants looked longer to the mouth of faces speaking in English or Spanish during infant-directed speech (but during adult-directed speech at 18 months only).

In sum, attention to the mouth supports language acquisition especially during sensitive periods spanning over the second half of the first year of life, with differences based on infants’ linguistic experience and ability to integrate auditory and visual information. Once infants are skilled enough in their native language they no longer focus more on the mouth unless visual and auditory information are not congruent, or the face speaks a foreign language. Given the relevance of mouth looking for language processing and learning, multiple questions should be raised about implication of face coverings during these sensitive periods. In particular, one could ask whether masks could affect acquisition of less familiar words or different accents, which would be even more relevant for bilingual populations.

##### Communication

When interacting with people wearing a mask, we realize that speech comprehension might be difficult, especially if we are speaking a language that is not our mother tongue. What about infants that are learning to decode the communicative meaning of speech without seeing lip and mouth movements? Will this impact their language development? To address these questions, we summarize the literature exploring the role of mouth processing for language development, in monolingual and multilingual environments.

The fact that the mouth region of a face is crucial for learning to communicate using verbal language is evident from studies of infants experiencing a multi-language environment. Comparing mono- and bilingual infants is useful to identify key aspects for the development of speech perception and comprehension skills, since only bilinguals need specific strategies to establish sounds, grammar, and social meaning of each of their languages (Werker and Byers-Heinlein, 2008). Differently from monolinguals, for bilinguals, equal attention toward eyes and mouth was found at 4 months and increased looking times toward the mouth were seen at 8 and 12 months, both while hearing their native and non-native language (Pons et al., 2015). Further, at 8 months, bilinguals can discriminate between two languages based on visual information only while this is not evident in monolinguals. Interestingly, this effect was found using languages infants had never been exposed to, suggesting the bilingual infants’ advantage generalizes to support new language processing (Sebastián-Gallés et al., 2012). Thus, these studies indicate that looking at the mouth is a crucial strategy to language learning, used from 8 months of age by infants who are exposed to multi-language contexts.

Timing of speech sounds and mouth movements involved appears crucial when it comes to detecting and disambiguating speech signals. Hillairet de Boisferon et al. (2017) found that at 10 months (but not at 4, 6, 8, and 12 months) infants looked more to the eyes in case of desynchronized speech, while they looked more to the mouth when audiovisual information was synchronized, both for native and non-native languages (Hillairet de Boisferon et al., 2017). The authors suggested that 10 month olds rely on eye information to disambiguate confusing linguistic information, while mouth looking is used for language learning when it provides useful visual cues (Hillairet de Boisferon et al., 2017). Although these findings also partly suggest that in the absence of coordinated audiovisual inputs language processing might be impacted, they are also somewhat encouraging with respect to possible compensatory effects of the eyes when the talking adult’s mouth is covered.

Exploring multisensory integration supporting speech learning, some authors investigated infants’ ability to match static articulatory configuration with produced sounds and found that this changes with age. For example, Streri et al. (2016) familiarized infants of 3, 6, and 9 months of age with faces producing hearable vowels while occluding the mouth and tested looking preference to pairs of full static images including the familiarization face. Infants looked longer to the congruent face at 3 months and to the incongruent face at 9 months, while no preference was manifested at 6 months. This suggests that infants’ ability to match audiovisual information for language learning consolidates close to 9 months of age (Streri et al., 2016). Of note, the type of sensory information available in the living context shapes how infants deploy attention to and integrate audiovisual cues. Mercure et al. (2019) compared visual scanning pathways of 4 to 8 month old during a McGurk task (see glossary on Table 3) and found that bimodal bilinguals (hearing infants of deaf mothers) do not shift their attention to the mouth as much as monolingual and unimodal bilingual infants do. From 6.5 months onward, bilinguals did not show a novelty preference when the auditory and visual information were not congruent, differently from monolinguals. The authors proposed that audiovisual speech experience is crucial for multi-modal speech processing (Mercure et al., 2019).

Notably, growing up infants and toddlers are more likely to find themselves in social interactive contexts whereby familiar and unfamiliar people interact with each other and not exclusively with them. Souter et al. (2020) found that 18 to 30 month olds prefer to look at the eyes rather than the mouth both when seeing a single actor singing nursery rhymes or talking infant-directed speech, and when multiple actors interacting with each other. Regarding multi-language exposure and conversations, Atagi and Johnson (2020) tracked infants’ gaze while seeing two women talking to each other and addressing the infant in a familiar or unfamiliar language. They found that bilinguals performed more anticipatory looks to talkers’ face when the language was unfamiliar rather than familiar. Thus, during challenging communicative events, different scanning patterns could be observed according to prior language exposure (Atagi and Johnson, 2020), highlighting that even consistent exposure to masked faces could have different effects on children’ language learning depending on their level of exposure to language.

In conclusion, beyond the first year of life, toddlers appear to focus on the mouth when entering the word acquisition phase of language development and then gradually shift again attention to eyes to complement language communicative meaning in function of their linguistic expertise. The differences in scanning strategies observed between monolingual and bilingual toddlers attending to conversations suggest that looking at the face is important when the spoken language is not familiar. Granting access to both visual and auditory speech information is crucial from 8 months of age, as infants make use of the synchronized sound and lip movement stimuli to learn a language. The analyzed literature suggests that face masks, which remove the visual mouth cue while probably muffling voice sounds, could have effects on language learning and understanding. Since children rely on facial cues to increase the amount of information that can help understanding the verbal content, we can expect conversations with masked faces to be more challenging for children who are less familiar with the spoken language.

#### Emotional Expressions

##### Perception

Facial expressions also have a central role in early learning; processing expressions require the use of configural information that is hindered by wearing face masks. To consider the potential impact of mask wearing on emotion processing, we describe studies examining its developmental underpinnings.

As discussed, newborns preferentially attend to faces, and especially dynamic faces. However, their ability to distinguish facial expressions is very limited. Newborns show novelty preferences when habituated to faces displaying dynamic changes in emotional expression regardless of the nature of the emotion (happiness and fear) (Leo et al., 2018). A facilitation effect of happy facial expressions is observed over the next months. At 3 months, happy facial expressions facilitate face recognition (Turati et al., 2011) when both eyes and mouth express happiness, but not in the case of happy eyes and an angry mouth or angry eyes and a happy mouth (Brenna et al., 2013). From this evidence, one could hypothesize that wearing a face mask would reduce or eliminate the facilitation effect of happy emotions for face recognition in early infancy because the mouth is not visible (*cf* Brenna et al., 2013). While these studies suggest that infants can discriminate between different emotional expressions from 3 months of age, others found that they need increased exposure to the emotional expressions (Flom et al., 2018) and the presence of multisensory cues (i.e., emotional voices; Flom and Bahrick, 2007) to show this ability before 5 months of age. Further research is needed to investigate whether the presence of auditory information might support face recognition despite the lack of information coming from the mouth, playing a compensatory role.

The emotional valence of faces has a key role in the development of face perception and learning abilities at later ages. At 6 months, happy emotional expressions increase infants’ preference for a face (Kim and Johnson, 2013) and at 7–8 months, rule learning is facilitated by happy expressions and disrupted by angry faces (Gross and Schwarzer, 2010; Quadrelli et al., 2020). By 8 months, infants not only can recognize changes in emotional expression and facial identity, but also they use these two pieces of information in conjunction for face recognition in upright faces, as suggested by a novelty preference based on emotional expressions independent of the face’s identity (Schwarzer and Jovanovic, 2010). Around this age, infants gradually learn to link auditory and visual emotional cues, and discrimination of the emotional valence of facial features becomes more refined. For instance, in 9-month-old infants, hearing emotional vocal sounds (laughing and grumbling) facilitates gaze shifts toward the face with a congruent facial expression paired with an incongruent face, providing evidence for a role of cross-modal top-down regulation on visual attention to facial expressions (Xiao and Emberson, 2019). The ability to integrate multisensory emotional cues seems to emerge only after the seventh month of age (Yong and Ruffman, 2016). It would be important to clarify whether emotion recognition is impoverished by mask wearing to understand whether it also affects a range of other domains.

The next developmental step includes the ability to discriminate between faces displaying different degrees of the same emotional expression. At 9 and 12 (but not 6) months, infants can discriminate faces along the happy-angry (but not happy-sad) continuum, while they are not able to discriminate variations within the same emotional category (Lee et al., 2015). These findings were interpreted as consistent with an infant inability to discriminate between faces within the same emotional category before the first year of life. This ability may develop in conjunction with emotion’s relevance for the infant. In fact, 6- to 7-month-old infants recognize subtle anger expressions when presented in a static but not dynamic face, suggesting they are sensitive to anger but possibly find it difficult to recognize it within a more dynamic context due to scarce experience of this emotion in their daily environment (Ichikawa et al., 2014; Ichikawa and Yamaguchi, 2014). On the contrary, subtle happy expressions are easier to be recognized in the presence of facial movements at the same age (Ichikawa et al., 2014). Considering the limited availability of facial cues due mask wearing that covers an important source of facial movement, infants might show less refined emotion recognition abilities for subtle emotional changes, especially for emotions that are less experienced in caregiving interactions.

Scanning strategies of faces in 7-month-old infants vary as a function of emotional expression. Segal and Moulson (2020a) showed that infants in general look more at the eyes than the mouth of fearful and happy faces presented side-by-side (Segal and Moulson, 2020a). An examination of infants’ looking time series revealed that they looked significantly longer to the eyes of angry and neutral faces, and to the mouth of happy faces in the first 3,000 ms, but scanning strategies were different for different emotional expressions (Segal and Moulson, 2020b). Interestingly, Geangu et al. (2016) showed that, while overall facial emotion recognition was found in both Western and East Asian 7-month-old infants, scanning strategies were different between the two groups, with Japanese infants looking more at the eyes and less at the mouth of happy and fearful faces compared to British infants. Importantly for the scope of the present review, these findings indicate that, while typical infants finally develop the ability to discriminate between emotional facial expressions, they might reach this milestone through different individual scanning strategies that are shaped by environmental exposure. In this view, we can expect infants who are predominantly presented with masked faces from 3 to 12 months of age to develop different strategies to process and interpret emotional expressions compared to infants who normally see the mouth as part of the emotional face configuration. However, given infants received normal full face exposure at home throughout the COVID-19 pandemic, it is also possible that they develop typical face scanning strategies when looking at non-masked faces.

##### Communication

Emotional expressions are used as communicative signals about the context. Fearful expressions might indicate the environment is threatening and are gradually prioritized by the infant’s attention system. While 3-month-olds seem to be greatly engaged by happy faces, by 5 months, a fearful attentional bias (Table 3) is observed. For example, attention disengagement from a central face toward a peripheral stimulus is slower for fearful than from a happy or neutral face (Peltola et al., 2008, 2011; Heck et al., 2016). When fearful and happy faces are presented side-by-side, increased attentional bias for the fearful face compared to happy and neutral faces is shown at 7–11 months, while at 5 month olds prefer happy faces (Peltola et al., 2009a, 2013). Of note, face familiarity does not affect fearful bias, as infants look longer to a novel fearful face when habituated to happiness, regardless of faces in the habituation phase being familiar or not (Safar and Moulson, 2017). From this evidence, it appears that the fearful attentional bias emerges toward the 7th month of age, despite a sensitivity to fearful faces can already be observed at 5 months.

To examine whether exposure to masked faces influences infants’ behavior and developmental processes elicited by the fearful bias, we need to know whether this is based on information derived from specific elements of the face or from the full facial configuration. Using artificially created faces, Peltola et al. (2009b) found longer latencies to disengage from fearful full faces but not fearful eyes alone at 7 months. The attentional bias to fearful faces at this age was associated with attachment security at 14 months of age, whereby infants who disengaged more easily from a fearful face in an overlap task showed more signs of attachment disorganization (Peltola et al., 2015). This finding corroborates the idea that early processing of emotional expression from the full face is involved in social development. This evidence appears particularly relevant when considering implications of mask wearing on emotion expression processing during development. According to Peltola et al. (2009b)’s results, eyes appear not to be sufficient for fearful bias to manifest at 7 months, possibly implicating that when wearing masks that leave only the eyes uncovered, fearful expressions might not elicit the same processes they would normally do, with potential cascading effects for later social development.

Importantly, preference for specific emotional expressions might vary depending on the individual infants’ temperamental characteristics as well as their parents’ emotional attitude (de Haan et al., 2004; Pérez-Edgar et al., 2017; Aktar et al., 2018; Fu et al., 2020). Highlighting the intertwin of individual temperament characteristics, caregiver affect dispositions, and the attentional bias toward certain facial expressions, this suggests that individual infants’ and caregivers’ temperament and affect dispositions may modulate the effect of mask wearing on the development of perceptual and communicative aspects of face processing.

Only in the second year, toddlers learn to distinguish between true and pretend emotional valence of the facial configuration. Walle and Campos (2014) examined 16- and 19-month-old behavioral responses to parental display of emotional expressions following a true or pretend distress situation. Parents were instructed to display pain and distress after perceptively hitting or missing their hand with a hammer. Both 16 and 19 month olds reacted with concerned facial expressions and prosocial responses more when they perceived the parents hit their hands (although only at 19 infants reacted with playful behavior and positive affect demonstrating they evaluated the context as playful; Walle and Campos, 2014). Further research will have to evaluate whether interacting with masked adults in times of COVID-19 has no effect on this the ability as emotional expression recognition skills have been acquired or whether mask wearing significantly limits toddlers’ experience to link emotional face configurations to contexts.

## Discussion

**Figure 2:**
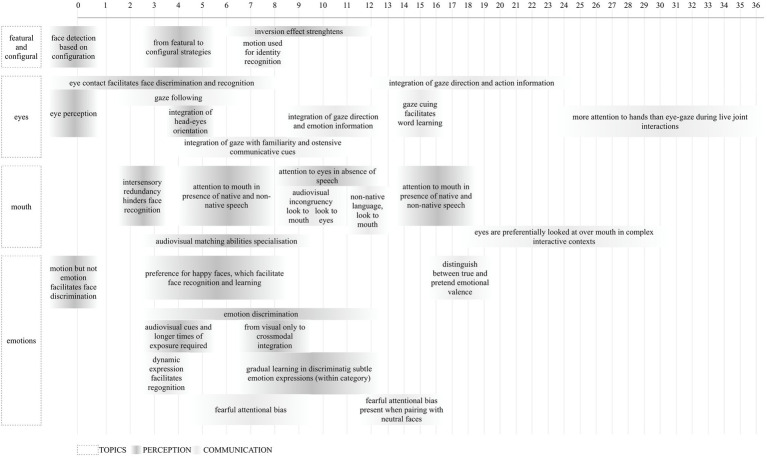
Age periods studied in the literature for each of the addressed topics.

**Figure 3:**
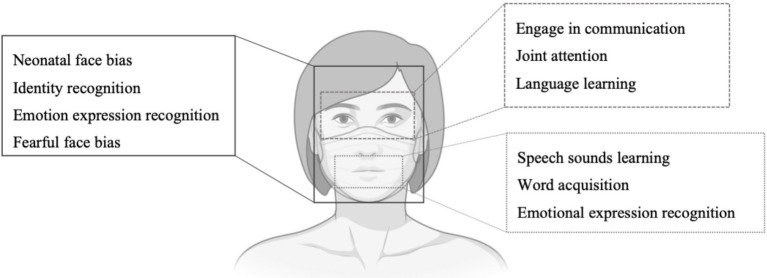
Psychological processes linked to face processing. Created with BioRender.com.

In the present work, we aimed to leverage the wide corpus of existing literature on sensitive periods for the specialization of face processing skills in early development (summarized in Figure 2) to generate hypotheses on possible effects of adults’ mask wearing adopted to limit COVID-19 diffusion. We asked which aspects of face processing might be altered by exposure to masked faces (Figure 3) and whether implications might differ as a function of infants’ developmental stage (main questions for future research emerged from the present review are summarized in Table 4).

**Table 4:** Outstanding questions.

Research topic	Discussed effect of face masks	Outstanding questions
FACE PROCESSING	Face masks hide the lower part of the face, possibly altering the infant’s perception of the face configuration.	Does augmented exposure to masked faces disadvantage face preference and the CONSPEC system during the first few months of life? Do the eyes compensate for limited exposure to the full facial information that was considered to be crucial for identity and emotional expression recognition?
AMOUNT OF EXPOSURE TO FULL FACES	Infants living in times of the COVID-19 are exposed to full faces in the home environment and through technological devices (i.e., tablets, smartphones, and TV), while they are more likely to be exposed to covered faces outside the home environment, including in child-care settings (depending on the country regulations).	Do scanning strategies infants used for identity and emotional expressions vary as a function of the amount of experience they have with faces wearing masks? Is exposure to un-masked faces within the home environment enough for infants to compensate for possible effects of the limited availability of some facial cues on identify and emotion recognition?
SOCIAL COMMUNICATION	By obscuring mouth dynamics, masks partly reduce the availability of communicative cues in a face while they leave eye information only available.	Could this influence face preference with effects of social engagement and joint attention? If so, is this effect age-specific? Could eye gaze compensate in those occurrences when mouth movements are not visually available?
SPEECH LEARNING	When the mouth is covered, audiovisual redundancy and information about mouth and lip movements for speech production that typically support speech learning during sensitive periods are missing.	Does this impact the speech sound learning and word acquisition, especially in multi-lingual contexts?

When investigating the potential impact of mask wearing on face processing during the first years of life, we need to differentiate according to individuals’ likelihood of being exposed to these stimuli. In fact, during the earliest stages of life, infants are more likely to spend most of the time within family contexts where they are not exposed to masked faces, while as they grow their daily environment includes people outside the household.

To discuss implications of mask wearing in infancy, it is crucial to describe how masks modify perceptual assets of faces. First, mask wearing disrupts configural face processing. When a mask is worn, no information can be obtained about the nose, cheeks, chin, mouth, and mouth movements. Second, processing of simultaneous changes in face features building up emotional expressions is limited due to the lower part of the face being covered. This limited exposure to facial configuration could possibly have implications in terms of both low-level perception and detection of communicative meanings. For this reason, developmental research in both areas has systematically been reviewed in the previous sections. Importantly, infants typically make use of multiple scanning strategies and pay differential attention to specific face regions and features to reach developmental milestones. Indeed, they gradually learn to analyze the eyes and gaze direction within the context of the entire face configuration—which contributes to the early face bias, identity recognition, as well as emotional expression discrimination—and they rely on audiovisual redundancy from others’ mouth for language learning. Thus, there could potentially be developmental effects if exposure to full faces is limited by widespread mask wearing.

### The Importance of the Full Face

Partially covering the face with a mask disrupts configural face processing, which largely constitutes the basis of facial discrimination and recognition abilities in adults. Developmental findings highlight that despite being sensitive to some configural variations as early as 3–5 months, infants clearly adopt configural scanning strategies around 7–8 months and master their use for upright face recognition toward the end of the first year. In recent studies with adults (Carragher and Hancock, 2020; Noyes et al., 2021) and children (Stajduhar et al., 2021), lower accuracy in identity and emotion recognition have been observed when processing masked faces. From this evidence along with that from developmental studies, we could hypothesize that similar effects could be found testing infants aged around 1 year of life. Moreover, because of the additional COVID-19 preventive measure of social distancing, unfamiliar faces might often be further away. Infants might then rely more on lower spatial frequency information of the face configuration because they cannot perceive details of featural characteristics. However, configural face processing is likely to be disrupted by the mask as well. Thus, social distancing may compound to mask wearing effects on identity and emotion recognition.

It should be noted that infants are not completely deprived of seeing full faces, which they normally encounter in the home environment. Further, since outside opportunities are reduced it is possible that infants living in COVID-19 times spend more time on technological devices where they are likely to be presented with a variety of full faces from streaming services and TV shows as well as videos, video calls on smartphones, and tablets, and other digital devices used by the older family members for socializing (Pandya and Lodha, 2021). In this respect, some suggestions might come from previous literature on monocular pattern deprivation during early development. For example, daily brief exposure to normal visual input greatly reduces the adverse effect of abnormal input due to monocular pattern deprivation during the sensitive period (Wensveen et al., 2006; Schwarzkopf et al., 2007). Given such findings, it is possible that the brief exposure to full faces infants are daily exposed at home throughout the COVID-19 pandemic may still be sufficient for the development of face recognition ability during infancy. However, the number of full faces infants are normally exposed to is reduced during the pandemic and, if the amount of faces they experience contributes to the development of perceptual and socio-communicative skills, some consequences might be observed in the next years. For such reasons, it is fundamental for future studies to explore developmental trajectories of face processing skills in infants born during COVID-19 pandemic accounting for the exposure they had to masked rather than full faces. Since masked faces are often experienced outside the family context, one prospective question concerns whether face processing will specialize more narrowly based on very familiar faces that infants see without masks. It is also possible that those who are highly exposed to masks adopt face recognition processes based on featural strategies. As patterned visual stimuli presented in the first month of life are necessary to initiate functional development of the visual neural pathways (Maurer et al., 1999), it would be important to know whether there are critical periods for exposure to certain visual stimuli in terms of configural face processing. Infant research focusing on face processing in children born in times of the COVID-19 pandemic should collect information about exposure to masked and full faces at the time of testing and possibly in the earlier stages of their life to control or test for effects of individual variability in masked face exposure on their key cognitive phenotype.

### Uncovered Eyes

Eye contact plays a crucial role in attention engagement, supporting face detection, and specialization of face processing skills from birth onward (Farroni et al., 2002, 2005; Johnson et al., 2015). While direct gaze facilitates face recognition and learning, gaze shifts coupled with head direction and other ostensive communicative signals scaffold the development of joint attention in the first semester of life. Toward the end of their first year, infants integrate gaze direction and emotion expression or hand actions to direct attention to the referred target, being able to rely on gaze cuing alone during the second year. Since the eye region is left uncovered by face masks, infants can access substantial socio-communicative information. Furthermore, masks could have the effect of driving attention to the eyes region. Relatedly, individuals who find focusing on the eye region or interpreting eye cues difficult [e.g., some autistic individuals (Senju and Johnson, 2009; Ashwin et al., 2015; Moriuchi et al., 2017; Pantelis and Kennedy, 2017)] could benefit from the exclusion of possibly competing visual information from the mouth region. Thus, attending the eyes region of the face might be easier in the presence of masked faces for these children from the first months of age, shaping developmental trajectories of social attention (Klin et al., 2015; Parsons et al., 2019). Alternatively, the mask could have a negative effect; for example, if they constitute an additional distractor. Further, masks may perhaps “force” attention to the eyes (the only visible feature), which may be associated with sensory over-stimulation for some people (Robertson and Baron-Cohen, 2017) and thus accelerate complete withdrawal from faces. Developmental longitudinal research is needed to test these hypotheses.

### Mouth for Language Learning

Infants further rely on facial information to learn language, by means of intersensory redundancy (Table 3) coming from mouth movements. They pay particular attention to the mouth between 4 and 8 months of age and gradually shift it to the eye region as their language expertise increases. After the first year, when entering the word acquisition phase, infants again pay selective attention to the interacting adults’ mouth to learn to articulate verbal sounds. If the speaking person has her mouth covered, infants cannot take advantage of audiovisual synchrony that is relevant for speech learning. A disadvantage linked to this could be particularly enhanced within multilingual environments, whereby infants rely on multisensory information to disentangle languages (Sebastián-Gallés et al., 2012; Pons et al., 2015). Sufficient experience with audiovisual information coupling during speech is required to exploit multi-modal speech processing in infancy (Mercure et al., 2019). Importantly, it should be noted that this experience might be acquired within the home environment with familiar adults and children. Further research is needed to elucidate whether partially transparent masks allow infants’ learning in contexts where masks are compulsory, assuming that linguistic stimulation within familial contexts can also play a compensatory role. From the published literature, we learn that bilingual infants make use of visual information coming from the mouth region to disambiguate between languages from 8 months of age. These infants could struggle more, particularly if they are mainly hearing the second language in community contexts (nursery, play-groups, and shops) where masks are used, and not as much at home.

Further compensation for language learning could be derived by eye contact and gaze following, which might foster language learning by directing infants’ attention to relevant cues in the environment (Çetinçelik et al., 2021). Thus, it appears important to investigate how much communicative content is vehiculated by facial features and cues beyond the mouth (i.e., eyes and head movements) and how to promote language learning more comprehensively. Crucially, the likelihood of exposure to masked faces, which intuitively increases with age as infants’ social environment broadens, needs to be considered when addressing these questions. In some countries, for example, face masks are mandatory among all adults within childcare settings; thus, the effects of mask wearing on infant development might be more important if the child spends a lot of time in these settings. Moreover, rules and guidelines might change within the same country depending on governmental decisions to face the COVID-19 pandemic, such that mask wearing might only impact development for a relatively short period of time. Studies investigating effects of mask wearing on development should consider and report these factors when selecting a study sample.

Another factor that may influence the effects of masks on face processing is the type of mask people wear, especially in childcare services. Plain-colored masks covering the mouth could foster attention to the eye region important for identity and emotion recognition as well as joint attention development. However, it is possible that very colorful masks direct infants’ attention away from the eyes, with the risk of limiting infants’ exposure to relevant social information. Transparent masks may allow infants to perceive orofacial movements while speaking and possibly enhance attention to the mouth region, reducing any risk of impacting language development. These factors should be considered by education and healthcare practitioners.

### Effects on Emotion Reading

Configural strategies also allow us to perceive and process emotion expressions. While a study on mask wearing effects shows that anger and happiness are discriminable in adults despite the covered mouth (Calbi et al., 2021), other findings also highlight difficulties in emotion reading more broadly due to mask wearing (Carbon, 2020; Noyes et al., 2021). Some authors argue that with masks becoming a common practice in everyday life, people have learnt to rely on eye information to discriminate emotions from masked faces, reflecting an adaptation of face processing secondarily to available visual information (Barrick et al., 2020). However, the perception of negative emotions produced by frowning was enhanced in adults when presented with masked emotional faces (Nestor et al., 2020). In infants, this possible bias toward negative interpretations of others’ expressions might have cascading effects on social communication. In this respect, it would be interesting to investigate emotional expression biases in infants exposed to masked faces during the COVID-19 pandemic and longitudinal effects of this on their own emotional development. During the second year of life, they rely on facial expressions in conjunction with their context (Walle and Campos, 2014). Whether not having access to configural information contribute to difficulties in emotion discrimination and understanding or whether, alternatively, the system specializes to allow processing based on alternative strategies is an interesting avenue for future research.

## Limitations

This review has some limitations. First, as for selection criteria, seminal research that hugely contributed to the field has not been discussed due to being published before 2000. We believe the content of such findings was reflected in following research included in the present review. Second, a selection bias might have occurred due to automatic filtering, which we tried to overcome by manually adding relevant literature cited in the included papers. Third, studies on atypical development of face processing, that has been largely investigated, were not included to limit the content of this review to papers investigating typical development of face processing. Future work should compare evidence from typical and atypical development to systematically delineate effects of mask wearing in the context of neurodiversity. Fourth, an important limitation concerns participation bias within studies that have been considered with Western or Asian countries being predominantly involved and participants recruited on voluntary basis. Studies of infants who are typically exposed to covered female faces due to religious reasons have not been found in our systematic search, but a cross-cultural comparison would have provided additional proofs about the possibilities proposed in our review. Last, while our search focuses on the first 3 years of life, we found that most studies pertain to infancy, suggesting that face processing is less investigated beyond the first year of life.

## Data Availability Statement

The original contributions presented in the study are included in the article/supplementary material, further inquiries can be directed to the corresponding author.

## Author Contributions

LC, AG, EJHJ, and TF contributed to the conception and design of the study. LC was primarily responsible for the literature search. LC and AG equally contributed to the article selection process, data extraction, and wrote the first draft of the manuscript. TF and EJHJ supervised the study. All authors contributed to manuscript revision and approved the submitted version.

## Funding

This study was funded by Beneficentia Stiftung Foundation to TF, by the ESRC grant no. ES/R009368/1 to AG and by the MRC Programme grant nos. MR/K021389/1 and MR/T003057/1.

## Conflict of Interest

The authors declare that the research was conducted in the absence of any commercial or financial relationships that could be construed as a potential conflict of interest.

## Publisher’s Note

All claims expressed in this article are solely those of the authors and do not necessarily represent those of their affiliated organizations, or those of the publisher, the editors and the reviewers. Any product that may be evaluated in this article, or claim that may be made by its manufacturer, is not guaranteed or endorsed by the publisher.
